# COVID-19-Related Giant Coronary Aneurysms in an Infant with Multisystem Inflammatory Disorder in Children: The First Case Report from the United Arab Emirates and the Arab Region

**DOI:** 10.1155/2021/8872412

**Published:** 2021-01-18

**Authors:** Ghassan Ghatasheh, Huda Al Dhanhani, Ashutosh Goyal, Muhammad Bassel Noureddin, Doaa Al Awaad, Ziad Peerwani

**Affiliations:** ^1^Division of Pediatric Infectious Disease, Tawam Hospital, Al Ain, UAE; ^2^Division of Pediatric Cardialgy, Tawam Hospital, Al Ain, UAE; ^3^General Pediatrics Department, Tawam Hospital, Al Ain, UAE; ^4^Clinical Laboratory Department, Tawam Hospital, Al Ain, UAE

## Abstract

**Background:**

Multisystem inflammatory disorder in children and adolescents is a relatively new and rare complication of COVID-19. This complication seems to develop after the infection rather than during the acute phase of COVID-19. The clinical features are similar to a well-known inflammatory syndrome in children, Kawasaki disease, and it can lead to collapse and multiple organ failure requiring intensive care. The COVID-19-associated multisystem inflammatory syndrome in children and adolescents is referred to mutually as pediatric inflammatory multisystem syndrome temporally linked with SARS-CoV-2 (PIMS-TS) or multisystem inflammatory syndrome in children (MIS-C) correlated with COVID-19, and here, it is referred to as MIS-C. *Case Presentation*. This report describes a nine-month-old Asian infant presented with a two-week history of fever with nonspecific signs of viral illness and erythematous rash. The clinical and biochemical findings were compatible with complicated typical Kawasaki disease (KD). The infant fulfilled the World Health Organization criteria for MIS-C and was treated with intravenous immunoglobulin and anticoagulation, which he responded well to. He was discharged home in good condition after almost 3 weeks of treatment.

**Conclusion:**

This case highlights a rare but new phenomenon attributed to severe acute respiratory syndrome coronavirus 2 infection. We report the first case report of MIS-C in the United Arab Emirates and Arab region. Among KD's complications, massive aneurysm with thrombosis is rare and usually will have deleterious results if not diagnosed and managed promptly.

## 1. Introduction

The impact of coronavirus disease 2019 (COVID-19) [1] has been widespread. Clinical presentations of severe acute respiratory syndrome coronavirus 2 (SARS-CoV-2) infection are milder in children than adults. The preliminary data centered on severe respiratory manifestations, which are seen predominantly in adults, amidst limited primary data on the burden of COVID-19 in children [2]. SARS-CoV-2 infection is frequently responsible for mild respiratory symptoms in children and adolescents compared to severe disease manifestations in adults [3, 4]. An association linking COVID-19 and late vasculitis manifestations has been increasingly speculated, especially in young asymptomatic patients, which may be due to postviral immunological responses [5, 6]. The clinical features of vasculitis are similar to a well-known inflammatory syndrome in children, Kawasaki disease.

A broader spectrum of MIS-C symptoms has been described. Notwithstanding differences in severity, coronary aneurysms occurred in all groups of patients [7]. Besides the broader clinical spectrum, there are various other distinguishing features of MIS-C compared with Kawasaki disease, including the age and ethnic background. Patients with MIS-C are typically greater than 7 years of African or Hispanic ancestry. A majority of patients with MIS-C present with an unusual cardiac injury defined by high troponin and brain natriuretic peptide levels, whereas others develop arrhythmia, left ventricle dysfunction, and significant coronary dilatation or aneurysms [8].

We describe the first case in the gulf region and United Arab Emirates (UAE) of multisystem inflammatory syndrome in children and adolescents related to COVID-19 with critical cardiac complications admitted to the general pediatric division of Tawam Hospital in Al Ain city, UAE.

## 2. Case Presentation

**Figure 1:**
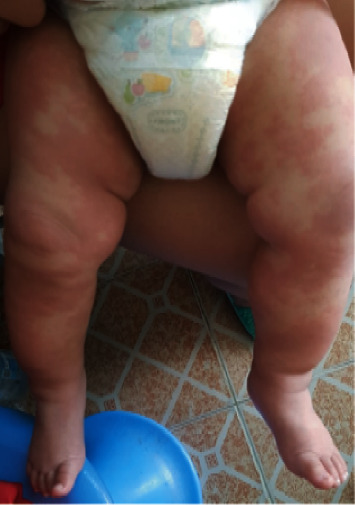
Erythematous rash over the lower limbs.

**Figure 2:**
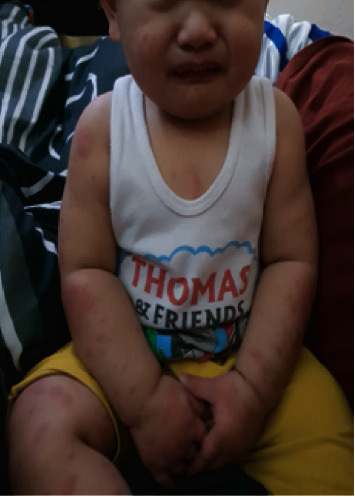
Rash over the entire body.

**Figure 3:**
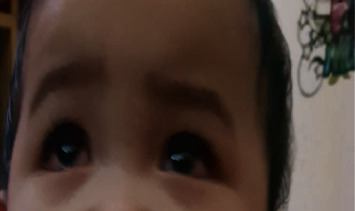
Bilateral nonpurulent conjunctivitis.

**Figure 4:**
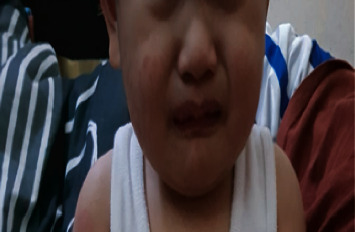
Dry cracked lips.

A nine-month-old Asian male from the Philippines presented to the emergency department with a two-week history of high-grade fever (up to 40°C) and skin rash. On the third day of fever, he developed an erythematous rash over the face and upper and lower extremities. He was diagnosed with foot mouth disease in a private health care facility and was managed with symptomatic treatment. The rash nearly disappeared by the end of the first week of illness; however, it progressed throughout the entire body, including the diaper area. The patient was thought to have an allergic reaction and was managed with antihistamines. Two days before his admission to our facility, he developed watery diarrhea 5–7 times/day, accompanied by decreased oral intake, excessive crying, swelling of hands and feet, skin peeling, and red eyes. On admission, he was alert, tired, and irritable. His vital signs revealed fever (40°C), respiratory rate of 30 breaths/min, heart rate of 130 beats/min, blood pressure of 92/47 mmHg, and normal oxygen saturation and capillary refill time. He had an erythematous rash over his entire body (face and upper and lower limbs) (Figures 1(a), 1(b), and 2), bilateral nonpurulent conjunctival injection (Figure 3), cracked lips (Figure 4), palpable small bilateral submandibular lymph nodes, and congested throat with hyperemic tonsils. The rest of the physical examination was nonrevealing.

A diagnosis of Kawasaki disease (KD) was made based on the above clinical features and paraclinical testing results. When asked, the parents denied any family history of Kawasaki disease from either parental sides. The patient received intravenous immunoglobulin (IVIG; 2 g/kg) and aspirin (100 mg/kg/day) divided over four doses. His fever subsided after 24 hours. Due to new reports describing an association between KD-like illness and SARS-CoV-2, a nasopharyngeal swab sample tested for SARS-CoV-2 by real-time PCR (RT-PCR) and COVID-19 serology test (Elecsys Anti-SARS-CoV-2 (Cobas)) was performed.

Interestingly, our patient tested negative for SARS-CoV-2 by PCR, but his serology tested positive for SARS-CoV-2-specific IgM, IgA, and IgG. Given these results, we elected to test his parents, and his mother tested positive for SARS-CoV-2 by RT-PCR.

**Figure 5:**
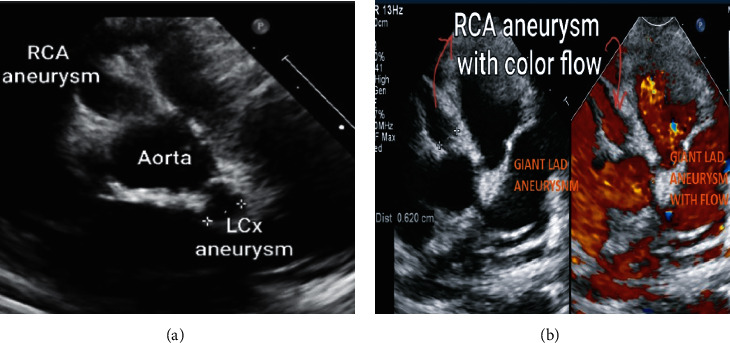
(a) Echocardiography still image showing left and right coronary aneurysms. (b) Echocardiography with color flow still image showing left and right coronary aneurysms.

The patient remained hemodynamically stable throughout, with no features of shock, cardiac failure, cardiac ischemia, or infarction. Troponin T serum levels remained within the normal range, and serial electrocardiograms did not show any pathological Q-wave or ST segment and T-wave changes, indicating an absence of cardiac ischemia or injury. However, the initial and serial follow-up echocardiogram (Figures 5(a) and 5(b)) showed a large aneurysm of the right coronary artery (RCA; 6.3 mm diameter with normalized *z* score ≥11) and left circumflex coronary artery (LCCA; 6 mm diameter with normalized *z* score >11). Moreover, a striking large aneurysm of the left anterior descending artery (LAD; cross-sectional diameter of 11 mm with *z* score >25) revealed multiple large and giant nonprogressive coronary aneurysms over the 4-week admission period.

Of note, there was good blood flow seen in the RCA aneurysm and distally into the RCA and LAD; otherwise, no cardiac abnormalities were noted. Ventricular function was preserved throughout the course of illness.

**Figure 6:**
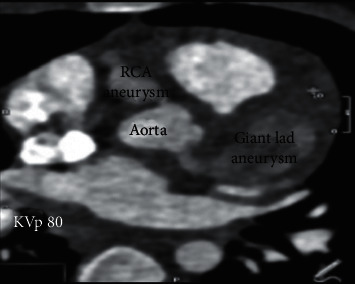
CT angiography showing massive LAD aneurysm.

The findings, including massive LAD aneurysm and other giant aneurysms, were further confirmed by the contrast-enhanced computed tomography (CT) angiogram (Figure 6). The left main artery is a standard caliber vessel, which gives rise to the LAD and circumflex arteries. The left main artery had no stenosis. Immediately after the bifurcation, the proximal LAD showed a large fusiform-type aneurysm (11.7 × 25.5 mm) with very faint opacification of the distal LAD. Aneurysms with multiple filling defects indicated contrast dilution or partial thrombus. The LCCA showed two small aneurysms (6.2 mm and 4.7 mm in diameter). No thrombus was noted in these two aneurysms. No aneurysms showed any calcification. Therefore, based on the multiple coronary artery aneurysms with filling defects/contrast dilution in the LAD and RCA, a diagnosis of KD was made.

As per the American Heart Association guidelines for patients at increased risk of thrombosis, for example, with large or giant aneurysms (≥8 mm or Z score ≥10) and a recent history of coronary artery thrombosis, “triple therapy” with ASA, a second antiplatelet agent, and anticoagulation with warfarin or LMWH may be considered [9]; therefore, the patient was treated with triple antithrombotic therapy given the high risk of occlusive thrombus and subsequent myocardial infarction.

The need for any cardiac intervention during this stage was discussed with pediatric cardiology experts both locally and internationally. Given the absence of features of cardiac ischemia or injury, it was agreed to withhold any cardiac surgical or interventional treatment options; however, the patient continued anticoagulation and antiplatelet therapy as per the KD guidelines.

The patient's clinical condition remained stable during the hospital stay, the international normalized ratio (INR) was checked frequently, and the LMWH dose was adjusted accordingly.

The patient was discharged in stable condition.

Unfortunately, further follow-up with the patient was lost due to insurance issues. However, we know that a pediatric cardiologist is following him up in the private sector and that a repeat echocardiogram still shows giant coronary aneurysms, but the patient's general condition is stable.

## 3. Investigation Laboratory Results

The investigation laboratory results are shown in Table 1. On admission, the patient remained febrile for 24 hours and tachycardic but was otherwise well appearing. A chest radiograph did not show cardiopulmonary anomalies. Given the 2-week history of fever, additional laboratory studies were conducted. Urinalysis revealed pyuria. Blood and urine cultures were collected as well as a nasopharyngeal PCR swab for common respiratory viruses and SARS-CoV-2. The patient had elevations in liver enzymes, ESR, and pro-brain natriuretic peptide (pro-BNP), but a normal troponin level. The following morning, echocardiogram and cardiac CT angiography were performed; a 12-lead ECG demonstrated no pathological findings. In light of few case reports linking COVID-19 with KD-like presentation, serologies for SARS-CoV-2 were sent one week after admission and on week 3 of illness. Once the patient serology reported as positive, both parents were screened for COVID-19 and only the patient's mother had tested positive for COVID-19 by nasopharyngeal PCR. The mother was never symptomatic, and she consequently screened negative on repeat testing. No other family members were tested positive, and all remained asymptomatic.

## 4. Discussion

To our knowledge, this is the first reported case of KD with concurrent COVID-19 infection in the gulf region and UAE as well as the first case of multisystem inflammatory disorder as per the World Health Organization's (WHO) case definition. KD was described in 1967 by Dr. Tomisaku Kawasaki [10] as a medium-sized vessel systemic vasculitis of unknown etiology [11]. KD prevalence is higher in Asian countries than in Western countries. The highest incidence rate is marked in Japan, followed by Korea and Taiwan, while lower rates are seen in Europe. It is regarded as the leading cause of acquired heart disease in children in developed countries, with 50% of cases happening in children <2 years old and 80% in children <5 years old [12]. The diagnosis of “classic” KD is considered in patients presenting pyrexia for five days concurrently with at least 4 out of 5 clinical criteria in the nonexistence of an alternative diagnosis.

Although KD's etiology prevails unclear, a viral trigger in some genetically predisposed children has been hypothesized [13]. A widely accepted hypothesis about the cause of KD implicates infectious agents as triggers to an inflammatory response, causing dysregulation in the host immune reaction in genetically predisposed individuals [14]. This study examined seasonal coronaviruses in some studies [15–18]. A group from Japan explored the association between two different coronaviruses (HCoV-NL63 and HCoV-229E) and KD by serological tests. The immunofluorescence assay detected no difference in HCoV-NL63 antibody positivity between patients and controls, whereas HCoV-229E antibody positivity was higher in patients with KD [19]. This suggests that the coronavirus family may represent one of the triggers of KD, with SARS-CoV-2 being a particularly virulent strain that elicits a robust immune response in the host. Given the disease pathogenesis, serology testing seems to be a more reliable tool than RT-PCR in detecting the cause of infection.

Angiotensin-converting enzyme 2 (ACE2) has been proved recently as the SARS-CoV-2 internalization receptor causing COVID-19 [20], in concert with the host's transmembrane protease serine 2 (TMPRSS2) membrane protease that primes the spike S protein of the virus to aid its cell entry [21]. The presence of TMPRSS2 significantly intensifies viral infectivity [22]. Protease inhibitors against TMPRSS2 appear to prevent viral entry and infection of lung cells in vitro.

TMPRSS2 and ACE2 assist SAR-CoV-2 entry, and the copresence of these 2 molecular substances in tissues to a large extent illustrates the tropism of viral proliferation. TMPRSS2 and ACE2 are coexpressed in many issues including the lung, heart, smooth muscle, liver, kidney, neurons, and immune cells [23].

The tissue localization of the receptors relates to COVID-19-presenting manifestations and organ dysfunction.

Vascular smooth muscle contains both ACE2 receptor and TMPRSS2 protease to aid viral entry and proliferation. Pathological examination of lung tissue and other affected organs has revealed evidence of microvascular inflammation concurrently with microvascular thrombi.

The presence of vasculitis and prothrombotic state resulted in increased pulmonary embolism frequency, which worsens hypoxemia by rising shunting in these already highly hypoxemic patients with acute respiratory distress syndrome. Despite all this evidence, our patient did not show any clinical evidence suggestive of pulmonary involvement, which parallels the observation that clinical features in symptomatic children are somewhat different from that in adults. Children tend to have more mild illness.

SARS-CoV-2 infection is often responsible for mild respiratory symptoms in children and adolescents, in opposition to severe forms reported in adults. However, novel reports from Europe and North America have described a new phenomenon affecting previously asymptomatic children with SARS-CoV-2 infection. It manifests as multiorgan involvement requiring admission to intensive care units, with some features similar to those of KD and toxic shock syndrome [24–28]. Besides the broader clinical spectrum, there are various other distinguishing features of MIS-C compared with Kawasaki disease, including the age and ethnic background. Patients with MIS-C are typically greater than 7 years of African or Hispanic ancestry. A majority of patients with MIS-C present with an unusual cardiac injury defined by high troponin and brain natriuretic peptide levels, whereas others develop arrhythmia, left ventricle dysfunction, and significant coronary dilatation or aneurysms [8].

The initial hypothesis is that this syndrome may be related to COVID-19 based on initial laboratory testing showing positive serology in most patients. Children are often treated with anti-inflammatory treatment, including parenteral immunoglobulin and steroids.

Our patient showed features comparable to those of the reported cases. However, few differences were noted. We are reporting a nine-month-old Asian male patient from the Philippines with KD and MIS-C features, while patients with MIS-C are typically older than 7 years and of African or Hispanic origin [8]. The reported cases are found to have marked lymphopenia, thrombocytopenia, and increased ferritin, as well as markers of myocarditis [24–28]. On the contrary, our patient had leukocytosis and thrombocytosis. Additionally, some patients had signs of severe disease, evidence of macrophage activation syndrome (MAS), resistance to IVIG, and need for adjunctive steroids [24–26]; our patient responded dramatically to one dose of IVIG.

The positive serology confirmed recent infection with SARS-CoV-2 since the qualitative antibody testing was reported to have a sensitivity of 95% and specificity of 85–90% when compared with PCR testing using a nasal swab. This finding, combined with IgG antibodies' positivity, suggests a late onset of the disease compared with the primary infection. More importantly, our case met the WHO case definition criteria of multisystem inflammatory disorder in children and adolescents [29]:

(1) Children and adolescents 0–19 years old with fever >3 days
(2) Any two of the following:
  1. Rash, bilateral nonpurulent conjunctivitis, or mucocutaneous inflammation signs (oral, hands, or feet)
  2. Hypotension or shock
  3. Features of myocardial dysfunction, pericarditis, valvulitis, or coronary abnormalities (including echo findings or elevated troponin or NT-pro-BNP)
  4. Evidence of coagulopathy (by PT, PTT, and elevated D-dimers)
  5. Acute gastrointestinal problems (diarrhea, vomiting, or abdominal pain)
(3) Elevated markers of inflammation, such as erythrocyte sedimentation rate, C-reactive protein, or procalcitonin
(4) No other apparent microbial causes of inflammation, including bacterial sepsis, staphylococcal, or streptococcal shock syndromes
(5) Evidence of COVID-19 (RT-PCR, antigen test, or serology positive) or likely contact with a COVID-19 patient

This case highlights a rare but new phenomenon attributed to SARS-CoV-2 infection. To the best of our knowledge, this is the first case report of MIS-C in the UAE and gulf region, as there have been limited data reported from the Middle East.

Although this patient may represent a usual presentation of KD outside of the SARS-CoV-2 epidemic, demographic and clinical management factors largely contributed independently to coronary aneurysm. The severity of cardiac manifestations with thrombi formation and evidence of SARS-CoV-2 infection in this case are significant findings when compared to children affected from other cohorts. SARS-CoV-2-infected children in these cohorts presented with PMI-C and features similar to those of KD and toxic shock syndrome. However, baseline electrocardiograms were nonspecific; a common echocardiographic finding was echo-bright coronary vessels (which progressed to a giant coronary aneurysm in one patient within a week of discharge from the pediatric intensive care) to mild dilatation of the coronary arteries [24, 25]. In addition, coronary artery events (thrombosis, stenosis, intervention, myocardial infarction, and death) occurred in 1% of patients with an aneurysm *z* score <10 and absolute dimension <8 mm, in 29% of patients with a *z* score ≥10 but an absolute dimension <8 mm, and in 48% of patients with both a *z* score ≥10 and absolute dimension ≥8 mm [9]. Our patient falls in the last category with up to 48% risk of serious coronary artery events in the future.

Algonaid et al. [30] reported the first case in KSA of a giant coronary aneurysm related to KD that persisted for five years, but fortunately with an uneventful course.

## 5. Conclusion

All these results and considerations support the hypothesis that the immune response to SARS-CoV-2 is responsible for a KD-like disease in susceptible patients. Given recent outbreaks of KD in Europe and North America and its association with recent SARS-CoV-2 infection, reporting new cases outside these geographic areas should prompt a high degree of vigilance among primary care and emergency physicians. Recognition of manifestations similar to KD is critical for timely treatment, which can have a significant impact on the preparedness and outcome in all countries while facing the COVID-19 pandemic.

## Figures and Tables

**Table 1:** Laboratory results.

	Event	Result	Reference range
6/5/2020, hospital admission day 1	Sodium level	133 mmol/L	136–145
	Potassium level	4.7 mmol/L	3.2–5.5
	Chloride level	94 mmol/L	98–107
	CO_2_	22 mmol/L	22–29
	Creatinine	22 *μ*mol/L	15–37
	Albumin level	24 g/L	35–52
	AST	97 IU/L	≤32
	ALT	62 IU/L	≤33
	WBC	24.1 × 10^9^/L	6.0–17.5
	RBC	4.09 × 10^12^/L	4.00–5.30
	Hgb	96 g/L	113–141
	Platelet	653 × 10^9^/L	140–400
	ESR	87 mm/hr	0–20
	C-reactive protein	110.0 mg/L	≤5.0
	Influenza A	Not detected	
	Influenza B	Not detected	
	Respiratory syncytial virus A	Not detected	
	Respiratory syncytial virus B	Not detected	
	Parainfluenza 1	Not detected	
	Parainfluenza 2	Not detected	
	Parainfluenza 3	Not detected	
	Parainfluenza 4	Not detected	
	Coronavirus OC43	Not detected	
	Coronavirus 229E	Not detected	
	Coronavirus NL63	Not detected	
	Coronavirus HKU1	Not detected	
	Adenovirus	Not detected	
	Human metapneumovirus	Not detected	
	*Mycoplasma pneumoniae*	Not detected	
	*Legionella pneumophila*	Not detected	
	2019-n-CoV RT-PCR	2019-nCoV not detected	
	Human bocavirus	Not detected	
	Human rhinovirus/enterovirus	Not detected	
	Influenza A/H1-2009	Not detected	
	Influenza A/H3	Not detected	
	Coronavirus (MERS-CoV)	Not detected	
	*Bordetella parapertussis* (IS1001)	Not detected	

7/5/2020	NT-pro-BNP	1,393.0 ng/L	0.0–320.0
	Troponin T	3.3 ng/L	≤14.0
	PT	10.3 s	9.5–12.5
	INR	0.93	0.87–1.15

11/5/2020	Troponin T	7.6 ng/L	≤14.0
13/5/2020, hospital admission day 7 3^rd^ week of illness	SARS-CoV-2 serology	Positive IgM, IgG, and IgA	
14/5/2020	Troponin T	16.7 ng/L	≤14.0
28/5/2020	Troponin T	15.2 ng/L	≤14.0
1/6/2020	PT	31.8 s	9.5–12.5
16/8/2020	INR	3.00	0.87–1.15

## Data Availability

All the data generated or analyzed during this clinical case report are included within this article.

## Abbreviations

COVID-19: Coronavirus disease 2019
KD: Kawasaki disease
IVIG: Intravenous immunoglobulin
MIS-C: Multisystem inflammatory syndrome temporarily associated with coronavirus
ECG: Electrocardiogram
RT: Reverse transcriptase
PCR: Polymerase chain reaction
RCA: Right coronary artery
LCCA: Left circumflex coronary artery
LAD: Left anterior descending artery
CT: Computed tomography
AHA: American Heart Association
WHO: World Health Organization
LMWH: Low-molecular-weight heparin
INR: International normalized ratio
TMPRSS2: Transmembrane protease serine 2
ACE2: Angiotensin-converting enzyme 2.

## References

[1] Coronaviridae Study Group of the International Committee on Taxonomy of Viruses (2020). The species severe acute respiratory syndrome-related coronavirus: classifying 2019-nCoV and naming it SARS-CoV-2. *Nature Microbiology*.

[2] Wang D., Hu B., Hu C. (2020). Clinical characteristics of 138 hospitalized patients with 2019 novel coronavirus- infected pneumonia in Wuhan, China. *JAMA*.

[3] Castagnoli R., Votto M., Licari A. (2020). Severe acute respiratory syndrome coronavirus 2 (SARS-CoV-2) infection in children and adolescents: a systematic review. *JAMA Pediatrics*.

[4] Dong Y., Mo X., Hu Y. (2020). Epidemiology of COVID-19 among children in China. *Pediatrics*.

[5] Galvan Casas C., Catala A., Carretero Hernandez G. (2020). Classification of the cutaneous manifestations of COVID-19: a rapid prospective nationwide consensus study in Spain with 375 cases. *British Journal of Dermatology*.

[6] Bouaziz J. D., Duong T., Jachiet M. (2020). Vascular skin symptoms in COVID-19: a French observational study. *Journal of the European Academy of Dermatology and Venereology*.

[7] Whittaker E., Bamford A., Kenny J. (2020). Clinical characteristics of 58 children with a pediatric inflammatory multisystem syndrome temporally associated with SARS-CoV-2. *JAMA*.

[8] Chiotos K., Bassiri E. M., Blatz A. M. (2020). Multisystem inflammatory syndrome in children during the coronavirus 2019 pandemic: a case series. *Journal of the Pediatric Infectious Diseases Society*.

[9] McCrindle B. W., Rowley A. H., Newburger J. W. (2017). A scientific statement for health professionals from the American Heart Association Diagnosis, Treatment, and Long-Term Management of Kawasaki Disease. *Circulation*.

[10] Kawasaki T. (2002). Pediatric acute febrile mucocutaneous lymph node syndrome with characteristic desquamation of fingers and toes: my clinical observation of fifty cases. *Pediatric Infectious Disease*.

[11] Mandai S., Pande A., Mandai D., Sarkar A., Kahali D., Panja M. (2012). Various coronary artery complications of Kawasaki disease: series of 5 cases and review of literature. *Journal of Cardiovascular Disease Research*.

[12] Rowley A. H., Shulman S. T. (2018). The epidemiology and pathogenesis of Kawasaki disease. *Frontiers in Pediatrics*.

[13] Turnier J. L., Anderson M. S., Heizer H. R., Jone P.-N., Glode M. P., Dominguez S. R. (2015). Concurrent respiratory viruses and Kawasaki disease. *Pediatrics*.

[14] Chen P.-S., Chi H., Huang F.-Y., Peng C.-C., Chen M.-R., Chiu N.-C. (2015). Clinical manifestations of Kawasaki disease shock syndrome: a case-control study. *Journal of Microbiology, Immunology and Infection*.

[15] Esper F., Shapiro E. D., Weibel C., Ferguson D., Landry M. L., Kahn J. S. (2005). Association between a novel human coronavirus and Kawasaki disease. *The Journal of Infectious Diseases*.

[16] Chang L.-Y., Lu C.-Y., Shao P.-L. (2014). Viral infections associated with Kawasaki disease. *Journal of the Formosan Medical Association*.

[17] Lehmann C., Klar R., Lindner J., Lindner P., Wolf H., Gerling S. (2009). Kawasaki disease lacks association with human coronavirus NL63 and human bocavirus. *The Pediatric Infectious Disease Journal*.

[18] Kim J. H., Yu J. J., Lee J. (2012). Detection rate and clinical impact of respiratory viruses in children with Kawasaki disease. *Korean Journal of Pediatrics*.

[19] Shirato K., Imada Y., Kawase M., Nakagaki K., Matsuyama S., Taguchi F. (2014). Possible involvement of infection with human coronavirus 229E, but not NL63, in Kawasaki disease. *Journal of Medical Virology*.

[20] Walls A. C., Park Y.-J., Tortorici M. A., Wall A., McGuire A. T., Veesler D. (2020). Structure, function, and antigenicity of the SARS-CoV-2 spike glycoprotein. *Cell*.

[21] Hoffmann M., Kleine-Weber H., Schroeder S. (2020). SARS-CoV-2 cell entry depends on ACE2 and TMPRSS2 and is blocked by a clinically proven protease inhibitor. *Cell*.

[22] Matsuyama S., Nao N., Shirato K. (2020). Enhanced isolation of SARS-CoV-2 by TMPRSS2-expressing cells. *Proceedings of the National Academy of Sciences*.

[23] Bertram S., Heurich A., Lavender H. (2012). Influenza and SARS-coronavirus activating proteases TMPRSS2 and HAT are expressed at multiple sites in human respiratory and gastrointestinal tracts. *PLoS One*.

[24] Riphagen S., Gomez X., Gonzalez-Martinez C., Wilkinson N., Theocharis P. (2020). Hyperinflammatory shock in children during COVID-19 pandemic. *Lancet*.

[25] Verdoni L., Mazza A., Gervasoni A. (2020). An outbreak of severe Kawasaki-like disease at the Italian epicentre of the SARS-CoV-2 epidemic: an observational cohort study. *Lancet*.

[26] Toubiana J., Poirault C., Corsia A. (2020). Outbreak of Kawasaki disease in children during COVID-19 pandemic: a prospective observational study in Paris, France.

[27] DeBiasi R. L., Song X., Delaney M. (2020). Severe COVID-19 in children and young adults in the Washington, DC metropolitan region. *Journal of Pediatrics*.

[28] Jones V. G., Mills M., Suarez D. (2020). COVID-19 and Kawasaki disease: novel virus and novel case. *Hospital Pediatrics*.

[29] https://www.who.int/news-room/commentaries/detail/multisystem-inflammatory-syndrome-in-children-and-adolescents-with-covid-19

[30] Algonaid O. A., Almoukirish A. S., Almashham Y. H. (2019). Giant right coronary artery aneurysm secondary to Kawasaki disease in an infant. *Journal of the Saudi Heart Association*.

